# Swedish trends in excision arthroplasty of the acromioclavicular joint during 2008-2023: a population-based register study

**DOI:** 10.1016/j.xrrt.2026.100751

**Published:** 2026-04-10

**Authors:** Viktor Mili-Schmidt, Kristofer Hallberg, Michael Axenhus

**Affiliations:** a Department of Orthopaedic Surgery, Danderyd Hospital, Stockholm, Sweden; b Department of Clinical Sciences at Danderyd Hospital, Karolinska Institutet, Stockholm, Sweden

**Keywords:** Clavicle, Incidence, Register study, Shoulder, Acromioclavicular joint, Excision arthroplasty, Distal clavicle resection

## Abstract

**Background:**

Excision arthroplasty of the acromioclavicular (AC) joint is a common procedure for degenerative, post-traumatic, and overload-related AC joint pathology; however, population-based data from Scandinavia are lacking. This study aimed to describe national trends in AC joint excision in Sweden from 2008 to 2023 and to forecast future incidence to 2040.

**Methods:**

This is an observational, population-based register study using the Swedish National Patient Register. All patients ≥15 years who underwent excision arthroplasty of the AC joint (Nordic Medico-Statistical Committee [NOMESCO] code NBG09) between January 1, 2008, and December 31, 2023, were included. Incidence rates per 100,000 inhabitants were calculated using annual population data and stratified by sex, age group, and region. Temporal trends were assessed with linear regression models and forecasts up to 2040.

**Results:**

In total, 41,555 patients underwent AC joint excision during the study period. Men consistently accounted for more procedures and had higher incidence rates than women. The incidence in men increased from 14.6 to 45.3 per 100,000 between 2008 and 2023, and in women from 10.4 to 32.6 per 100,000. The highest incidence was observed in individuals aged 50-69 years. All regions exhibited rising incidence over time. Forecast modeling indicated a continued increase in procedure incidence through 2040.

**Conclusion:**

The incidence of AC joint excision arthroplasty in Sweden has risen steadily since 2008 and is projected to continue increasing. These findings highlight a growing surgical burden and underscore the need for evidence-based guidelines, clinical outcome studies, and cost-effectiveness analyses to optimize indications and reduce unwarranted treatment variation.

The acromioclavicular (AC) joint plays a critical role in shoulder girdle biomechanics, contributing to scapulothoracic rhythm and facilitating overhead and cross-body movements.^5^^,^^7^

Due to high axial loads across its relatively small contact area, the AC joint is prone to degenerative change over time, especially in individuals exposed to repetitive overhead activity, heavy lifting, or high-demand physical work.^21^ In addition to degenerative osteoarthritis, AC joint pathology may arise from traumatic injury, most commonly AC dislocation or distal clavicle fracture, which can lead to chronic instability and post-traumatic changes.^11^^,^^14^ Osteolysis of the distal clavicle is another important cause of AC joint pain, classically observed in weightlifters.^2^^,^^22^

Pathology of the AC joint is a frequent cause of shoulder pain and can result in substantial functional limitations and reduced quality of life.^10^^,^^21^ Nonoperative treatment, including activity modification, physiotherapy, nonsteroidal anti-inflammatory medication, and corticosteroid injections, represents the primary management strategy for AC joint pathology.^10^^,^^21^ When conservative treatment fails, surgical intervention may be indicated.^10^^,^^21^ Excision arthroplasty of the distal clavicle, originally described by Mumford and later modified using arthroscopic techniques, is a surgical procedure intended to relieve pain by eliminating pathological contact between the distal clavicle and the acromion.^13^ Both open and arthroscopic distal clavicle resection have been reported to provide reliable pain relief and high patient satisfaction in appropriately selected cases.^15^

Epidemiologic studies have also documented a growing overall utilization of AC joint excision.^12^ McLean et al^12^ reported that in an urban population, the annual incidence of AC joint excision doubled from 9.3 to 19.6 per 100,000 between 2009 and 2013, with approximately 85% of cases being performed arthroscopically in the later years of that period. Despite these insights from other countries, there is a paucity of data on AC joint excision trends in Scandinavia. Understanding national trends in AC joint excision is essential to contextualize changes in surgical practice and to evaluate potential shifts in indications, patient demographics, and health care utilization. In publicly funded health care systems, procedure incidence also has implications for resource allocation and future surgical workforce planning.

The objective of the present study was to investigate national trends in AC joint excision arthroplasty in Sweden from 2008 through 2023, including procedure incidence over time stratified by sex, age, and region, and to forecast future trends to 2040.

## Materials and methods

### Study design setting

This is an observational population-based register study. The data are open access and publicly available from the National Patient Register (NPR). This study adheres to the REporting of studies Conducted using Observational Routinely-collected health Data (RECORD) guidelines.

Sweden is a high-income nation with a well-developed economy and health care system. Its health care model is founded on the principle of universal, utilitarian access for all Swedish citizens and permanent residents. In addition, citizens of other European Union countries are entitled to health care services under bilateral agreements. Oversight of the health care system is provided by the Swedish National Board of Health and Welfare.

Health care services in Sweden include emergency care, hospital treatment, and outpatient visits. A defining feature of the system is that health care is free at the point of delivery. Both public and private sectors operate within the national framework. Every resident of Sweden is assigned a permanent personal identification number, which is used in all interactions with public and private health care providers. This system ensures consistency and integration in health care registration and data management across all national health registers.

### Data source

NPR is a nationwide database that contains information on patients who have received care within the inpatient health care system, as well as those treated by physicians in outpatient settings. Reporting to the NPR is mandatory for all health care providers. The register has been in operation since 1964 and achieved nationwide coverage in 1987. Specialized outpatient care data were incorporated in 2001.^4^

Quality assessments have shown that the data in the NPR are most reliable from 2008 onward. Prior to that year, registration was incomplete; however, subsequent improvements have largely addressed these gaps. The registry is updated monthly, with correctional data incorporated as needed.

Information recorded in the NPR includes surgical procedures coded according to the NOMESCO classification system, as well as data on geographic distribution, age groups, and sex.^25^ Each patient is counted once per year, per code, and per region, meaning that if a patient receives the same NOMESCO-coded procedure multiple times in a year, only the first instance is recorded. The NPR also contains diagnostic information coded using the International Statistical Classification of Diseases, Tenth Revision.^26^

### Patients

#### Inclusion criteria

Individuals aged 15 years or older at the time of surgery who were resident in Sweden during the procedure were included. Eligible individuals underwent excision arthroplasty of the AC joint, defined by the NOMESCO code NBG09. Surgeries performed between January 1, 2008, and December 31, 2023, were included.

#### Exclusion criteria

Individuals residing outside Sweden at the time of surgery were excluded, as well as those who underwent arthrodesis or other surgical procedures involving the AC joint.

### Variables

Incidence data were stratified by age group (15-49, 50-59, 60-69, 70-79, and ≥80 years), sex, and region of performed surgery.

### Statistics

Descriptive statistics were presented as counts and percentages. Incidence rates were calculated per 100,000 inhabitants using year-specific population data obtained from Statistics Sweden. To avoid duplicating entries, each unique personal identification number was included only once per calendar year, surgical procedure, and geographic region.

Annual procedure counts and incidence rates per 100,000 inhabitants were summarized with 95% confidence intervals. Temporal trends in incidence were evaluated using linear regression models, with year as the independent variable. Models were fitted separately for men and women, and results are reported as slopes (β) with corresponding 95% confidence intervals.

Forecasts for 2024-2040 were generated using models fitted to data from 2008 to 2023 and are presented as point estimates with 95% prediction intervals where applicable. All analyses were performed using R version 4.5.2 (stats package) and Python 3.11 (scikit-learn 1.4, pandas 2.2, matplotlib 3.8).

### Ethics

The study was performed using open-source data and was therefore not subject to ethical review.

## Results

### Descriptive data and patients

**Figure 1:**
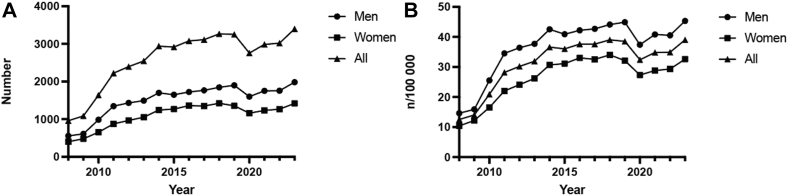
The total number of procedures (**A**) and the incidence (**B**) of procedures per sex during 2008-2023.

A total of 41,555 patients were identified between 2008 and 2023. Overall, men had a higher total number of procedures (Fig. 1*A*) and a higher incidence rate (Fig. 1*B*) compared with women (Table I).

**Table I:** Incidence of excision arthroplasty in the AC joint per 100.000 per sex and year.

Sex	2008	2009	2010	2011	2012	2013	2014	2015	2016	2017	2018	2019	2020	2021	2022	2023
Men	14.6	15.9	25.5	34.5	36.4	37.7	42.5	40.9	42.2	42.7	44.1	44.9	37.4	40.8	40.5	45.3
Women	10.4	12.2	16.5	22.0	24.1	26.2	30.7	31.1	33,0	32.5	34,0	32.1	27.3	28.8	29.3	32.6

*AC*, acromioclavicular.

**Figure 2:**
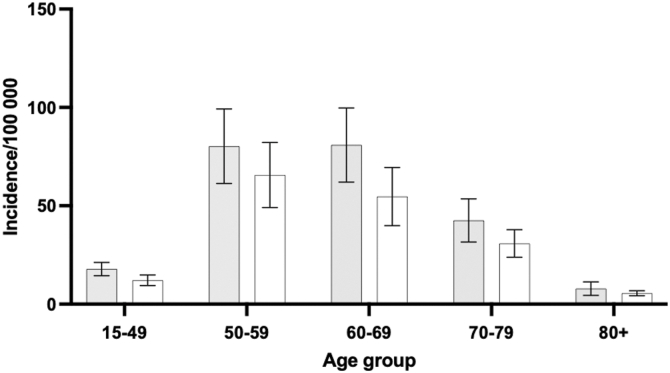
Age-group stratification of procedures per sex, with the average during 2008-2023 shown. White indicates men and gray indicate women. Error bars are 95% CI. *CI*, confidence interval.

When stratified by age, the highest incidence was observed in the 50-59 and 60-69 year age groups, while younger (15-49) and older (≥70) groups showed lower incidence rates (Fig. 2).

**Figure 3:**
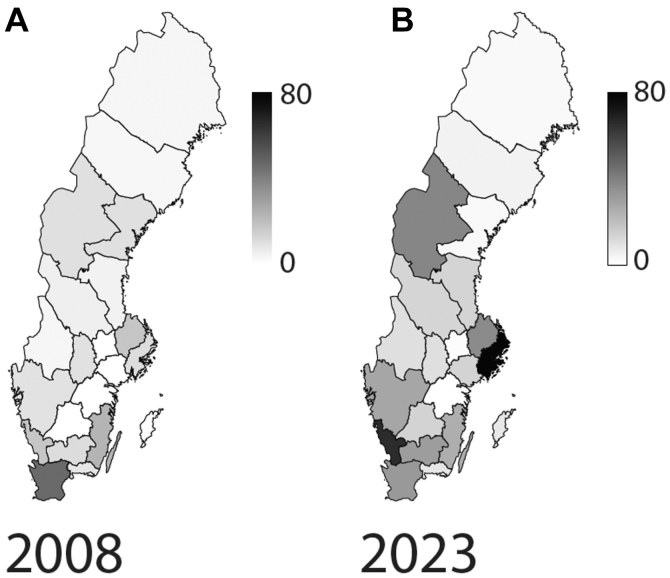
Regional disparities in procedural incidence during 2008 (**A**) and 2023 (**B**). Incidence per 100,000 inhabitants.

Regional analysis demonstrated a low overall incidence in 2008 (Fig. 3*A*), with only one region showing a noticeable concentration of procedures. By 2023, the incidence had increased across all regions, with several showing a particularly high rate (Fig. 3*B*).

**Figure 4:**
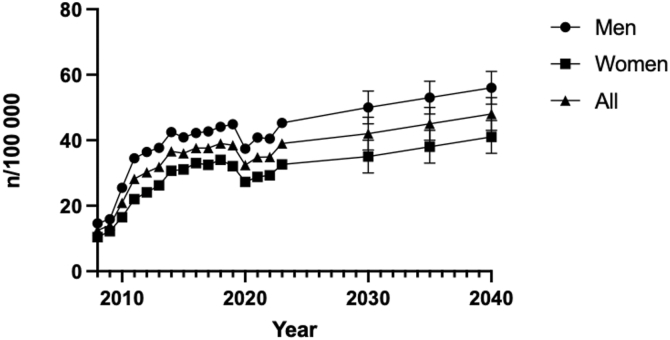
Predicative forecast of surgery trends per sex up until 2040. Error bars indicate 95% CI. *CI*, confidence interval.

Forecast modeling predicts that the incidence of excision arthroplasty will continue to rise through 2040 (Fig. 4). The projected increase is most pronounced among men.

## Discussion

This register study is, to our knowledge, the first comprehensive assessment of temporal and demographic trends in excision arthroplasty of the AC joint in Sweden. The study found that incidence of excision arthroplasty has increased steadily between 2008 and 2023, with projections indicating continued growth through 2040. The rise was observed across all geographic regions, with the highest incidence in middle-aged adults and with consistently higher rates in men than women.

The higher incidence observed among men may reflect greater exposure to occupational and recreational activities associated with repetitive overhead loading and traumatic AC joint injuries,^3^^,^^16^^,^^18^ including contact sports, heavy labor, and military service, populations in which men remain disproportionately represented. Similarly, the peak incidence in the 50-69 year age groups align with the expected progression of degenerative joint changes and the delayed consequences of earlier shoulder trauma.^17^^,^^20^

Regional variation observed in the current study suggests potential differences in surgical practice patterns, referral behavior, or resource availability. This is not surprising given the lack of national guidelines for excision arthroplasty indication in Sweden. Earlier studies on other shoulder procedures have reported geographical and national disparities, highlighting the influence of local clinical culture and health care access.^8^^,^^9^ Further research may help clarify whether these variations reflect differences in disease burden or surgical indication thresholds.

The observed long-term increase in procedure volume may be driven by several factors, including increased public awareness and patients demanding surgical treatment. The trend is noteworthy, as there is a paucity for evidence of the superiority of surgical treatment compared to nonoperative treatment.^23^^,^^24^

Importantly, although excision arthroplasty is widely performed and generally associated with favorable outcomes, evidence remains scarce regarding optimal surgical technique and patient selection.^6^^,^^19^^,^^24^ As the procedure becomes increasingly common, research is needed to refine indications, evaluate long-term results, and verify that surgical treatment is a cost-effective treatment option.

The increase observed in Sweden exceeds that reported in previous population-based studies. In an urban Scottish population, McLean et al^12^ reported that the incidence of AC joint excision increased from 9.3 to 19.6 per 100,000 between 2009 and 2013. In comparison, the Swedish incidence rose from 14.6 to 45.3 per 100,000 in men and from 10.4 to 32.6 per 100,000 in women between 2008 and 2023, representing approximately a threefold increase in both sexes and substantially higher absolute rates in recent years. A study from the United States has similarly demonstrated increasing utilization of distal clavicle excision, primarily driven by arthroscopic techniques.^1^ Although direct comparison should be interpreted cautiously because of differences in health care systems, case definitions, and denominators, these findings suggest that the upward trend observed in Sweden reflects a broader international increase in the use of distal clavicle excision.

A major strength of the present study is the use of a national, mandatory reporting register with near-complete coverage, minimizing selection bias and enabling population-level analysis. The large sample size allows for robust trend evaluation and future forecasting. However, several limitations must be considered. The NPR does not include granular clinical data such as indication for surgery, imaging findings, operative technique, or post-operative outcomes. Coding inaccuracies and the inability to account for repeat surgeries, bilateral procedures, and staged surgeries may also influence estimates. Finally, forecasts of future trends rely on historical patterns and may be affected by future changes in health care policy, treatment algorithms, or surgical technology.

## Conclusion

The incidence of excision arthroplasty of the AC joint is increasing steadily in Sweden since 2008. These findings suggest a growing surgical burden and potential differences in local indications and practice patterns in the absence of national guidelines. Given the limited evidence supporting the superiority of surgery over nonoperative treatment and the lack of consensus regarding optimal technique and patient selection, the continued expansion of AC joint excision underscores the need for high-quality clinical studies and cost-effectiveness analyses. Development of evidence-based guidelines may help harmonize indications, reduce unwarranted treatment variations, and ensure that patients most likely to benefit are appropriately selected for surgery.

## Disclaimers:

Funding: No funding was disclosed by the authors.

Conflicts of interest: The authors, their immediate families, and any research foundations with which they are affiliated have not received any financial payments or other benefits from any commercial entity related to the subject of this article.

## Availability of data

The data sets can be obtained from the NPR directly (https://www.socialstyrelsen.se/en/statistics-and-data/statistics/statistical-databases/).
